# Healthy orthorexia vs. orthorexia nervosa: Italian validation of the Teruel Orthorexia Scale (TOS)

**DOI:** 10.1007/s40519-023-01568-x

**Published:** 2023-05-03

**Authors:** Giorgio Falgares, Giulia Costanzo, Giovanna Manna, Daniela Marchetti, Juan Ramón Barrada, María Roncero, Maria Cristina Verrocchio, Sonia Ingoglia

**Affiliations:** 1 Department of Psychology, Educational Sciences and Human Movement, University of Palermo, Palermo, Italy; 2 Department of Psychological, Health and Territorial Sciences, University “G. d’Annunzio” of Chieti-Pescara, Chieti, Italy; 3 Departamento de Psicología y Sociología, Universidad de Zaragoza, Teruel, Spain; 4 Departamento de Personalidad, Evaluación y Tratamientos Psicológicos, Universitat de València, Valencia, Spain

**Keywords:** Orthorexia nervosa, Healthy orthorexia, Italian validation, Teruel Orthorexia Scale, Eating disorders

## Abstract

**Purpose:**

Orthorexia nervosa (OrNe) is a potentially pathological condition characterized by a fixation on healthy diet. An increasing number of studies have been conducted on this mental preoccupation, but the validity and reliability of some of the psychometric instruments employed in its assessment are still under debate. Among these measures, the Teruel Orthorexia Scale (TOS) seems to be promising, given that it allows to differentiate between OrNe and other non-problematic forms of interest with healthy eating, named as healthy orthorexia (HeOr). The aim of this study was to examine the psychometric properties of an Italian version of the TOS, by testing its factorial structure, internal consistency, test–retest reliability, and validity.

**Method:**

Through an online survey, we recruited 782 participants from different regions of Italy, asking them to complete the following self-report instruments: TOS, EHQ, EDI-3, OCI-R, and BSI-18. From the initial sample, 144 participants agreed to complete a second TOS administration 2 weeks later.

**Results:**

Data confirmed the validity of the 2-correlated factors structure of the TOS. The questionnaire also showed good reliability, both in terms of internal consistency and temporal stability. With regard to the TOS validity, results showed that OrNe was significantly and positively associated with measures of psychopathology and psychological distress, while HeOr showed no correlations or negative associations with the above-mentioned measures.

**Conclusion:**

Based on these results, the TOS can be considered a promising measure for the assessment of both pathological and non-problematic forms of orthorexic eating behavior also in Italian population.

**Level of evidence:**

Level V, descriptive cross-sectional study.

## Introduction

The term orthorexia comes from the Greek words “*orthós*”, meaning “right”, and “*òrexsis*”, signifying “appetite”. It was introduced by Bratman in 1997 [1] to describe a pathological fixation on healthy food and proper nutrition, named as orthorexia nervosa (OrNe). OrNe implies ritualized patterns of eating, whose transgression induces important negative effects such as anxiety, shame, feelings of guilt, and an exaggerated sense of personal impurity, which can eventually lead the individual to punish themselves [2, 3]. These obsessive food-related behaviors can lead to clinically significant consequences, including impairments in important areas of psychological functioning and medical complications [4, 5].

Although a consensus on the definition and diagnostic criteria of OrNe has recently been published [6], studies are still needed to clarify its relationship with other pathological conditions (above all, eating disorders and obsessive–compulsive disorder) [7–9]. Indeed, some of the psychometric measures traditionally employed in the evaluation of OrNe present important methodological concerns, possibly affecting the validity of previous findings on the characteristics of OrNe and its associations with other variables [10].

Up to now, the most employed instruments to assess OrNe are the ORTO-15 [11], the Eating Habits Questionnaire (EHQ) [12], and the Düsseldorf Orthorexia Scale (DOS) [13].

The ORTO-15 was developed in Italy by Donini et al. in 2005 [11]. It has been validated in different languages [14–19], representing the most popular measure used in OrNe assessment. It is a self-report questionnaire made-up of 15 items that investigate three different areas: cognitive-rational area, clinical area, and emotional area. These items are rated on a 4-point Likert scale, ranging from *always* to *never*; higher total scores are indicative of normal eating behaviors, while lower total scores highlight the presence of orthorexic behaviors. Even though the ORTO-15 represents the most used instrument to evaluate OrNe, its psychometric properties seem to be inadequate at different levels: it shows low internal consistency and doubtful content validity, and its internal structure seems to be inconsistent across several samples (for a review, [18]). Also, it may overestimate the prevalence of OrNe because of its inability to differentiate normative dieting from pathological eating behaviors [10].

Another self-report questionnaire is the EHQ, ideated by Gleaves et al. in 2013 [12]. It consists of 21 items that explore beliefs related to healthy eating, feelings associated with it, and problems related to healthy eating behaviors; these dimensions are investigated through three different subscales: *Knowledge*, *Feelings*, and *Problems*. All items are rated on a 4-point Likert scale, ranging from 1 (*false, not at all true*) to 4 (*very true*). The original version of the EHQ has good internal consistency and test–retest reliability, as well as adequate convergent, discriminant, and criterion-related validity. However, according to Roncero et al. [18], it also presents some limitations, such as the absence of items considering negative emotions associated with OrNe (i.e., shame, guilt, sadness, etc.).

Finally, Barthels et al. developed the DOS in 2015 [13]. It is a tool composed of 10 items rated on a 4-point Likert scale, ranging from 1 (*this does not apply to me*) to 4 (*this applies to me*). Total scores higher than 30 indicate the presence of OrNe, while scores between 25 and 29 are indicative of risk for OrNe. This instrument shows good psychometric properties (Cronbach’s alpha = 0.84; test–retest correlation *r* = 0.70), but apparently it does not allow to differentiate between orthorexic and anorexic behaviors in patients with Anorexia Nervosa [20].

Besides the above-mentioned limitations, all these measures have one characteristic in common: their internal structure seems unclear, given that there is preliminary evidence indicating the presence of items also assessing non-problematic forms of interest in healthy dieting [i.e., “The way my food is prepared is important in my diet” (EHQ), “Eating the way I do gives me a sense of satisfaction” (EHQ), “I have certain nutrition rules that I adhere to” (DOS)] [21]. This can lead to confusing results, in which harmless and non-problematic approaches to healthy eating may be erroneously pathologized and mistaken for indicators of OrNe [22].

The need to clearly distinguish between a pathological dimension of orthorexia and a non-problematic interest in healthy eating has been recently pointed out by Barrada and Roncero [23], who have proposed to define these two dimensions as, respectively, OrNe and healthy orthorexia (HeOr). While HeOr can be defined as an healthy interest in diet, (self-assessed) healthy behaviors with regard to diet, and eating healthily as part of one’s identity [23], the core element of OrNe is a strong preoccupation with healthy diet with negative emotional, cognitive, and/or social consequences while trying to approach this goal and when the eating behavior deviates from it. Importantly, HeOr is not a equivalent of healthy eating and, thus, should not be confused with it [24]. Specifically, individuals scoring high on HeOr report that healthy eating is an important part of their life and that they spend time and energy to healthy eating-related activities. However, their beliefs about what should be considered as healthy dieting may not coincide with an objective or real definition of healthy eating. Also, not all the people with high diet quality (according to external standards, not self-definition) consider their diet to be so, nor do all of them consider their diet a relevant aspect of their identity. Thus, healthy diet and HeOr can and should be differentiated.

The introduction of the HeOr construct has provided theoretically relevant information that has allowed to reconceptualize the way OrNe is conceived. For example, it may be misleading to consider investing time in cooking or planning healthy meals as a symptom of OrNe, given that being highly engaged in an activity cannot be in itself a problematic behavior. Consequently, referring to HeOr also when the research focus is primarily on OrNe may help to overcome the risk to pathologize neutral or even positive activities and interests.

Barrada and Roncero [23] have developed a new instrument, the Teruel Orthorexia Scale (TOS), a 17-item self-report measure that assesses OrNe, including its cognitive, emotional, and social components, and HeOr. They found that while OrNe is associated with obsessive–compulsive symptoms, disordered eating styles, and other pathological conditions, HeOr seems to be independent from psychopathology, or inversely associated with it.

This two-dimensional questionnaire showed adequate psychometric properties, with good internal consistency (Cronbach’s alpha > 0.80) and adequate test–retest reliability over an 18-month period (*r* > 0.70) for both factors. The correlation between OrNe and HeOr was moderate (*r* = 0.43), which indicates that these two dimensions cannot be considered as parts of the same continuum, ranging from people who are not interested in healthy eating to individuals excessively preoccupied with it (OrNe), passing to people mildly interested in healthy nutrition (HeOr).

The TOS has been validated in other languages, such as French-Canadian [25], English-American [22, 26], Portuguese [27], Arabic [28], and Turkish [29], showing also in these countries adequate factorial validity, reliability, and measurement invariance as a function of participants characteristics. These studies have also supported the TOS convergent validity, showing positive associations between OrNe and eating disorder symptoms [22, 25, 26, 29], obsessive–compulsive symptoms [26, 29], emotional distress [25, 26, 29], and perfectionism [26], and to a lesser extent between HeOr and eating disorder symptoms [25, 26]. Moreover, they found that HeOr was unrelated to obsessive–compulsive symptoms and perfectionism [26], negatively related or unrelated to emotional distress [26, 29], and positively related to positive affect [29].

## The present study

Based on this background, the TOS could be a promising measure, which can contribute to better defining the boundaries between a healthy and protective behavior and a problematic condition. Consequently, it may represent a useful instrument in clinical practice, leading to more accurate assessments and targeted interventions.

For these reasons, the general aim of our research study was to further examine the psychometric properties of the TOS by contributing to the Italian validation of this self-rating scale. This can contribute to broaden the conceptualization of orthorexia also in the Italian context, by evaluating the holding of the bidimensional structure of this measure in our culture. Specifically, the first aim was to investigate the TOS factorial structure testing a series of models. The *1-factor model* assumes a unidimensional structure, in which each item provides a measure of a single construct; it was tested to reject the hypothesis of unidimensionality of the orthorexic construct. The *2-correlated factors model* hypothesized by Barrada and Roncero [23] specifies two different and related dimensions of orthorexia: HeOr and OrNe. We expected that results would confirm the adequacy of the 2-correlated factors structure also in the Italian cultural context (Hp1).

The second aim was to test its reliability, which was examined both in terms of internal consistency and test–retest over a 2-week interval. We expected that the TOS would show adequate reliability, in line with previous validations in other languages and countries (Hp2).

The third aim was to investigate its validity examining the relation of OrNe and HeOr with symptoms of orthorexia measured by the EHQ, psychological distress dimensions (expressed in terms of anxiety, somatization, and depression), obsessive–compulsive symptomatology, and eating disorders symptoms (expressed in terms of eating disorder risk, affective problems, and overcontrol). With regard to symptoms of orthorexia measured by the EHQ, although the EHQ internal structure seems unclear, we considered relevant to include this measure in our study for a better comprehension of the characteristics of these instruments. We hypothesized that:

- orthorexic symptoms measured by the EHQ would be positively associated with both HeOr (Hp3a) and OrNe (Hp3b), as prior findings evidenced that EHQ items seem to tap both OrNe and HeOr dimensions [21];
- anxiety, somatization, and depression would be unrelated or negatively associated with HeOr (Hp4a) and positively with OrNe (Hp4b);
- obsessive–compulsive symptomatology would be unrelated or negatively associated with HeOr (Hp5a) and positively with OrNe (Hp5b);
- eating disorder risk, affective problems, and overcontrol would be unrelated or negatively associated with HeOr (Hp6a) and positively with OrNe (Hp6b).

## Method

### Participants

We used an online survey to recruit 782 Italian adults (82% female) ranging in age between 18 and 75 years (*M*_age_ = 32.3, SD = 11.55) from different regions of Italy. To participate in the study, respondents had to be at least 18 years old and had to be able to read and understand Italian. From the initial sample, 144 participants (88.2% female), ranging in age between 20 and 55 years (*M*_age_ = 25.2, SD = 5.88), agreed to complete the TOS for a second time 2 weeks later (18% of the sample), provided contact information and completed the second administration. Socio-demographic information is reported in Table 1.

**Table 1:** Socio-demographics

	Initial sample *n* = 782	Re-test sample *n* = 144
	% or *M* (SD)	% or *M* (SD)
Gender		
Female	82	88.2
Male	18	11.8
Age	32.3 (11.55)	25.2 (5.88)
Educational level		
Middle school diploma	1.9	0
High school diploma	36.3	25.7
Undergraduate degree	46.4	74.3
Postgraduate degree	15.3	0
Income		
Low	42.7	51
Medium–low	37.5	31.5
Medium–high	15.6	10.5
High	4.1	7
Marital status		
Single	41.8	44.4
Engaged	27.9	47.9
Married/cohabitating	26.2	6.9
Separated/divorced	4	0.7
Widowers	0.1	0
Diet^a^		
Yes	29.9	28.5
No	70.1	71.5
Physical activity		
Yes	51	41
No	49	59

^a^Low-carbohydrate, low-fat, high-protein, vegetarian, vegan, or Mediterranean diet

### Procedure

The Ethical Committee of the Department of Psychological, Health and Territorial Sciences at G. d’Annunzio University of Chieti-Pescara (protocol number 21010) approved the study and all procedures were performed in accordance with the ethical principles for psychological research, following the Declaration of Helsinki and its revisions [30] as well as the ethics guidelines of the American Psychological Association [31].

Participants were recruited from April 15, 2020, to January 25, 2021, using an online survey developed via Qualtrics, whose link was shared via email and on various platforms such as social media and websites. The first page of the survey included information regarding the research aims, the voluntary nature of the participation, and the anonymity of responses. Consent was requested before proceeding with the data collection. Participants did not receive any form of compensation for their participation.

### Measures

#### Socio-demographics

Respondents were asked to indicate their gender, age, and educational qualification, as well as their socioeconomic level and marital status. Moreover, respondents were also asked to indicate if they were following a diet and if they regularly practiced physical activity.

#### Teruel Orthorexia Scale

Orthorexia was assessed with the Teruel Orthorexia Scale (TOS) [23]. It is a 17-item self-report measure articulated in 2 subscales: healthy orthorexia (9 items, “*My interest in healthy food is an important part of the way I am, of how I understand the world*”), which indicates a healthy, non-pathological interest in proper nutrition, and orthorexia nervosa (8 items, “*I feel overwhelmed or sad if I eat food that I consider unhealthy*”), which represents an extreme preoccupation with healthy diet that may lead to relevant emotional, social, and cognitive impairments. All items are rated on a 4-point Likert scale, ranging from 0 (*completely disagree*) to 3 (*completely agree*). The Italian version of the TOS was developed using the back-translation method [32]. First, a bilingual researcher translated the instructions and items from the original Spanish version into Italian. Next, the Italian version was back-translated into Spanish by another bilingual researcher to confirm whether the translation matched the wording of the original scale. Finally, the original and the back-translated versions were compared. When discrepancies occurred between the two versions, the researchers worked together to make corrections to the Italian version. No items were eliminated or significantly adjusted during the translation process.

#### Eating Habits Questionnaire-21

The 21-item Eating Habits Questionnaire (EHQ-21) [12] is a self-report measure that explores (a) beliefs related to healthy eating (via the “Knowledge” subscale), (b) feelings associated with healthy eating (via the “Feelings” subscale), and (c) problems related to these behaviors (via the “Problems” subscale). Each item is rated on a 4-point Likert scale, ranging from 1 (*false, not at all true*) to 4 (*very true*). As we have noted in the introduction, the internal structure of the EHQ is not completely clear, but we have included this measure in the study for completeness. In the current study, the internal reliability consistency value, as indexed by Cronbach’s *α*, were 0.86 for Feelings, 0.89 for Knowledge, and 0.91 for Problems.

#### Brief Symptom Inventory-18

Symptoms of psychological distress were assessed with the Brief Symptom Inventory-18 (BSI-18) [33]. It is a 18-item self-report measure articulated in 3 subscales: Somatization, Anxiety, and Depression. Items are rated on a 5-point Likert scale, ranging from 0 (*not at all*) to 4 (*extremely*). In the current study, the subscales had adequate internal consistency, with Cronbach’s α equals to 0.76 for Somatization, 0.87 for Anxiety, and 0.84 for Depression.

#### Obsessive–Compulsive Inventory—Revised

Obsessive–compulsive symptomatology was assessed with the Italian version of the Obsessive–Compulsive Inventory—Revised (OCI-R) [34, 35]. It is an 18-item self-report measure articulated in 6 subscales: Washing, Checking, Obsessing, Hoarding, Ordering, and Mental Neutralizing. Items are rated on a 5-point Likert scale, from 0 (*not at all*) to 4 (*extremely*). In the present study, the subscales had adequate internal consistency, with Cronbach’s α values ranging from 0.66 for Checking and Mental Neutralizing to 0.89 for Obsessing.

#### Eating Disorder Inventory-3

Symptoms and psychological features of eating disorders were assessed with the Italian version of the Eating Disorder Inventory—third edition (EDI-3) [36, 37]. It is a 91-item self-report scale articulated in 12 subscales, consisting of 3 eating-disorder-specific subscales (Drive for Thinness, Bulimia, and Body Dissatisfaction), and 9 general psychological subscales (Low Self-Esteem, Personal Alienation, Interpersonal Insecurity, Interpersonal Alienation, Interoceptive Deficits, Maturity Fears, Perfectionism, Asceticism, and Emotional Dysregulation). All items are rated on a 6-point Likert scale, ranging from 1 (*never*) to 6 (*always*). In the present study, the subscales had adequate internal consistency with Cronbach’s *α* values ranging from 0.67 for Asceticism to 0.91 for Drive for Thinness.

### Data analysis approach

Preliminarily, we computed descriptive statistics (means, standard deviations, skewness, and kurtosis) to investigate the TOS items distribution. We also computed Pearson correlation coefficients in order to examine their interrelations.

As a first step, in order to investigate the TOS factorial validity, we run a series of Confirmatory Factor Analysis (CFA), testing the hypothesized 2-correlated factors model against the 1-factor model. Due to the non-normal distribution of some items, the adjusted Weighted Least Squares Means and Variance (WLSMV) estimation method was used since it is the more suitable procedure to use for CFA with ordered categorical factor indicators [38]. The 2-correlated factors model was evaluated, allowing each item to load on the hypothesized factor and setting all other factor loadings at zero; factor covariances were free parameters to be estimated; to establish the measurement scale of each factor, their variance was fixed at 1.0. Following Zickgraf and Barrada [22], who found that, although the TOS internal structure was clear and theoretically interpretable, secondary loadings cannot be expected to be equal to zero, we also run Exploratory Structural Equation Models (ESEM) [39]. In cases like this, a CFA would distort the recovered parameters [22, 40, 41]. For the ESEM, we used target rotation. To statistically evaluate the closeness of the hypothetical model to the empirical data, multiple goodness-of-fit indexes were used, including the Comparative Fit Index (CFI), the Root Mean Square Error of Approximation (RMSEA), and the Weighted Root Mean Square Residual (WRMR). The Chi-square test of model fit was not used as an evaluation of absolute fit because of its sensitivity to sample size. CFI values ≥ 0.90, RMSEA values ≤ 0.08, and WRMR values < 1 were interpreted as evidence of acceptable fit to the data, CFI values ≥ 0.95 and RMSEA values ≤ 0.05 were interpreted as evidence of excellent fit to the data [42, 43]. Analyses were performed using Mplus 7 [44].

As a second step, to examine the TOS reliability, we run a series of analysis based on the best fitting CFA model. We examined the coefficient *R*^2^ to evaluate the single item reliability indicator [45]: *R*^2^ values ≥ 0.50 can be considered acceptable [46]. We used the composite reliability indicator *ρ*_c_ to evaluate the reliability of the constructs as it has been considered a better indicator than Cronbach’s alpha [45]; values ≥ 0.70 can be considered acceptable [47]. Finally, in order to examine test–retest reliability, we computed the Intraclass Correlation Coefficient (ICC) [48, 49].

As a third step, in order to investigate the TOS validity, we run four separate ESEMs with latent variables examining the associations of HeOr and OrNe as measured by the TOS with (a) orthorexic symptoms (EHQ), (b) dimensions of psychological distress, specifically somatization, anxiety, and depression, (c) obsessive–compulsive symptomatology, and (d) eating disorders symptoms, specifically eating disorder risk, affective problems, and overcontrol. In the first model, orthorexic symptoms (EHQ) was specified as a latent variable measured by the EHQ composite scores of knowledge, feelings, and problems. In the second model, somatization, anxiety, and depression were specified as latent variables measured by items of the respective BSI-18 subscales. In the third model, obsessive–compulsive symptomatology was specified as a latent variable measured by the OCI-R composite scores of hoarding, checking, ordering, mental neutralizing, washing, and obsessing. In the last model, (a) eating disorder risk was specified as a latent variable measured by the EDI-3 composite scores of drive for thinness, bulimia, and body dissatisfaction, (b) affective problems was specified as a latent variable measured by the EDI-3 composite scores of interoceptive deficits and emotional dysregulation, and (c) overcontrol was specified as a latent variable measured by the EDI-3 composite scores of asceticism and perfectionism. To establish the measurement scale of each factor, their variance was fixed at 1. The fit of these models was assessed using the goodness-of-fit indexes described above.

## Results

### Preliminary analyses

Means, standard deviations, skewness, and kurtosis of the TOS items, and Pearson correlation coefficients are reported in Table 2. Data had a normal univariate distribution, the skewness and kurtosis values being approximately in the range from − 1.0 to + 1.0 [50], with the exception of items 5 (“*My social relations have been negatively affected by my concern about eating healthy food*”), 14 (“*I avoid eating with people who do not share my ideas about healthy eating*”), 16 (“*If, at some point, I eat something that I consider unhealthy, I punish myself for it*”), and 17 (“*Thoughts about healthy eating do not let me concentrate on other tasks*”), all referring to OrNe, which showed a non-normal distribution. Correlation coefficients varied from 0.01 to 0.58.

**Table 2:** Means (*M*), standard deviations (SD), skewness (*S*), and kurtosis (*K*) of the TOS items, and Pearson correlation coefficients

	1	2	3	4	5	6	7	8	9	10	11	12	13	14	15	16	17
TOS1	–																
TOS2	0.46	–															
TOS3	0.37	0.56	–														
TOS4	0.26	0.25	0.17	–													
TOS5	0.10	0.22	0.13	0.31	–												
TOS6	0.36	0.55	0.52	0.25	0.27	–											
TOS7	0.36	0.41	0.43	0.25	0.21	0.53	–										
TOS8	0.40	0.57	0.56	0.18	0.12	0.56	0.52	–									
TOS9	0.28	0.45	0.33	0.40	0.43	0.51	0.39	0.40	–								
TOS10	0.24	0.30	0.25	0.48	0.34	0.38	0.33	0.30	0.54	–							
TOS11	0.27	0.36	0.32	0.15	0.08	0.36	0.34	0.37	0.29	0.31	–						
TOS12	0.25	0.26	0.16	0.59	0.36	0.34	0.27	0.20	0.52	0.58	0.20	–					
TOS13	0.36	0.46	0.44	0.26	0.16	0.51	0.57	0.53	0.39	0.40	0.36	0.33	–				
TOS14	0.07	0.22	0.15	0.19	0.27	0.25	0.23	0.16	0.33	0.30	0.11	0.32	0.23	–			
TOS15	0.24	0.39	0.38	0.19	0.14	0.43	0.38	0.38	0.40	0.31	0.28	0.23	0.38	0.24	–		
TOS16	0.07	0.13	0.01	0.36	0.42	0.22	0.09	0.07	0.38	0.35	0.03	0.50	0.13	0.31	0.15	–	
TOS17	0.08	0.08	0.02	0.25	0.41	0.18	0.14	0.04	0.36	0.24	0.06	0.34	0.13	0.28	0.10	0.49	–
*M*	2.41	1.51	1.38	1.33	0.28	1.16	0.97	1.59	0.68	1.05	1.70	0.81	1.22	0.16	0.58	0.23	0.13
SD	0.67	0.88	0.93	1.02	0.65	0.94	0.91	0.85	0.83	0.91	0.91	0.93	0.92	0.47	0.81	0.59	0.43
Skewness	− 0.91	− 0.05	− 0.02	0.19	2.60	0.39	0.59	− 0.19	0.98	0.46	− 0.27	0.92	0.33	3.25	1.26	2.98	3.99
Kurtosis	0.50	− 0.70	− 0.91	− 1.09	6.52	− 0.76	− 0.55	− 0.54	0.00	− 0.68	− 0.70	− 0.12	− 0.74	11.11	0.82	9.21	18.09

Correlation coefficients ≥ 0.10 were significant at *p* < 0.05

### The TOS factorial structure

To test the hypothesized factorial structure of the TOS, three models were run: (a) a 1-factor model, in which all items loaded on a single factor; (b) a 2-factor CFA model, in which items loaded on the factor where they theoretically belong; and (c) a 2-factor ESEM, equivalent to an Exploratory Factor Model, in which all items loaded on all factors. Goodness-of-fit indexes are reported in Table 3. Results showed that the 1-factor model had not a good fit to the data, the 2-factor CFA had a good fit to the data, and the 2-factor ESEM had an excellent fit to the data being CFI > 0.95 (Hp1). The standardized solution of the 2-factor ESEM is shown in Table 4. The examination of standardized estimates of factor loadings reveals that estimated parameters were substantial (ranging from 0.53 to 0.89), robust standard errors were small (ranging from 0.02 to 0.04), and t values were high and significant (ranging from 15.37 to 45.24). Only item 9 (“*My concern with healthy eating takes up a lot of my time*”), designed to assess orthorexia nervosa, had a high and significant cross-loading.

**Table 3:** Goodness-of-fit indexes of models testing the TOS factorial structure and those testing the relation of the TOS dimensions with other study variables

	*χ*^2^	*df*	*p*	CFI	RMSEA	RMSEA 90% CI	WRMR
Models testing the factorial structure of the TOS							
1-factor model	1777.75	119	< 0.001	0.837	0.134	0.128–0.139	2.981
2-factor CFA model	653.32	118	< 0.001	0.947	0.076	0.071–0.082	1.747
2-factor ESEM model	321.62	103	< 0.001	0.979	0.052	0.046–0.059	0.948
Models testing the relations of the TOS with other study variables							
Orthorexic symptoms (EHQ)	981.54	167	< 0.001	0.924	0.079	0.074–0.084	1.81
Psychological distress dimensions	1077.49	535	< 0.001	0.972	0.036	0.033–0.039	1.115
Eating disorders symptoms	611.39	227	< 0.001	0.963	0.047	0.042–0.051	1.075
Obsessive–compulsive symptoms	559.17	212	< 0.001	0.968	0.046	0.041–0.050	1.178

**Table 4:** Standardized factor loadings of the ESEM 2-factor model of the TOS

Italian version	English version	Healthy orthorexia	Orthorexia nervosa
TOS1. Mi sento bene quando mangio cibo sano (HeOr)	TOS1. I feel good when I eat healthy food (HeOr)	**0.60**	0.04
TOS2. Dedico molto tempo all’acquisto, alla pianificazione e/o alla preparazione di cibo, affinché la mia alimentazione sia più salutare possibile (HeOr)	TOS2. I spend a lot of time buying, planning, and or/preparing food so my diet will be as healthy as possible (HeOr)	**0.79**	− 0.01
TOS3. Credo che il mio modo di mangiare sia più salutare di quello della maggior parte delle persone (HeOr)	TOS3. I think that my way of eating is healthier than that of most people (HeOr)	**0.82**	− 0.17
TOS4. Mi sento in colpa quando mangio qualche alimento che considero non sano (OrNe)	TOS4. I feel guilty when I eat food that I do not consider healthy (OrNe)	0.01	**0.72**
TOS5. Le mie relazioni sociali sono state influenzate negativamente dalla mia preoccupazione per il cibo sano (OrNe)	TOS5. My social relationships have been negatively affected by my concern about eating healthy food (OrNe)	0.05	**0.68**
TOS6. Il mio interesse per l’alimentazione sana è una parte importante del mio modo di essere e di comprendere il mondo (HeOr)	TOS6. My interest in healthy food is an important part of the way I am, of how I understand the world (HeOr)	**0.73**	0.13
TOS7. Preferisco mangiare un alimento sano e poco gustoso, piuttosto che un alimento gustoso e non salutare (HeOr)	TOS7. I’d rather eat a healthy food that is not very tasty than a good-tasting food that isn’t healthy (HeOr)	**0.71**	0.04
TOS8. Mangio principalmente cibo che considero sano (HeOr)	TOS8. I mainly eat foods that I consider healthy (HeOr)	**0.89**	− 0.15
TOS9. La mia preoccupazione per l’alimentazione sana occupa molto del mio tempo (OrNe)	TOS9. My concern with healthy eating takes up a lot of my time (OrNe)	**0.40**	**0.56**
TOS10. Mi preoccupa la possibilità di mangiare alimenti poco salutari (OrNe)	TOS10. I am concerned about the possibility of eating unhealthy foods (OrNe)	0.22	**0.62**
TOS11. Non mi importa di spendere di più per acquistare un alimento, se credo che questo sia più salutare (HeOr)	TOS11. I don’t mind spending more money on a food if I think it’s healthier (HeOr)	**0.53**	0.01
TOS12. Mi sento triste o sopraffatto/a se mangio cibo che non considero sano (OrNe)	TOS12. I feel overwhelmed or sad if I eat food that I consider unhealthy (OrNe)	0.01	**0.86**
TOS13. Preferisco mangiare poco e sano, piuttosto che saziarmi con cibo che potrebbe non essere salutare (HeOr)	TOS13. I would rather eat a smaller portion of healthy food than get full from food that may not be healthy (HeOr)	**0.70**	0.08
TOS14. Evito di mangiare con persone che non condividono le mie idee sull’alimentazione sana (OrNe)	TOS14. I avoid eating with people who do not share my ideas about healthy eating (OrNe)	0.22	**0.51**
TOS15. Provo a convincere le persone che mi circondano a seguire le mie abitudini alimentari sane (HeOr)	TOS15. I try to convince the people in my life to follow my healthy eating habits (HeOr)	**0.59**	0.08
TOS16. Se qualche volta mangio cibo che considero non sano, mi punisco per questo (OrNe)	TOS16. If I ever eat something I consider unhealthy, I punish myself for it (OrNe)	− 0.22	**0.93**
TOS17. I pensieri sull’alimentazione sana non mi permettono di concentrarmi su altre attività (OrNe)	TOS17. Thoughts about healthy eating prevent me from concentrating on other tasks (OrNe)	− 0.18	**0.84**

*HeOr* healthy orthorexia, *OrNe* orthorexia nervosa

Factor loadings ≥ |0.19| were significant at *p* < 0.05. In bold, factor loadings ≥ 0.30

### The TOS reliability

**Fig. 1:**
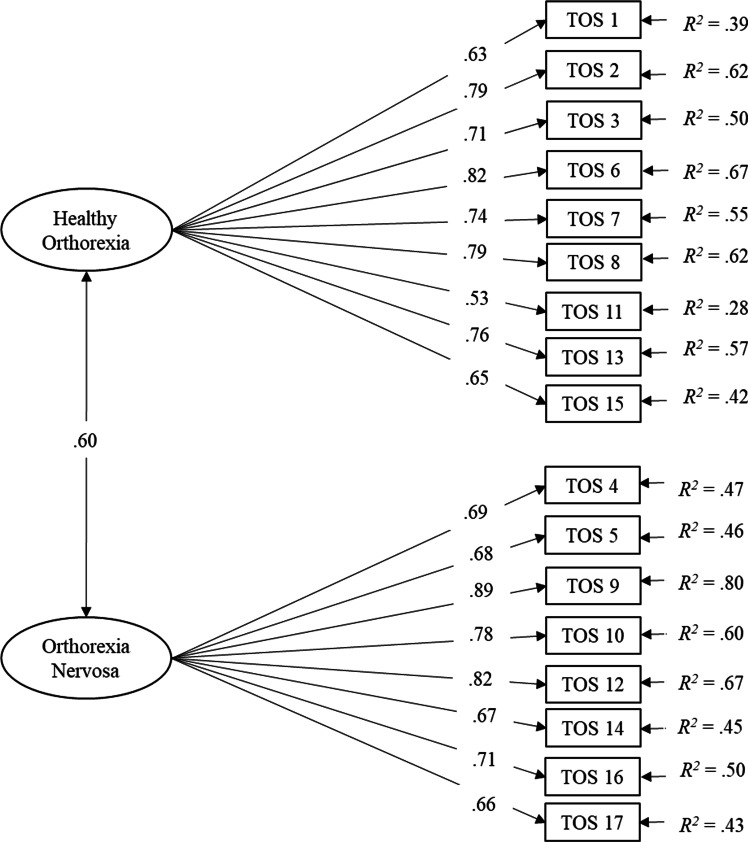
The 2-factor CFA model of the TOS. Standardized solution. All parameter estimates are significant at *p* < 0.05

The single item reliability indicators (*R*^2^) computed on the basis of results of the 2-factor CFA model are reported in Fig. 1. Results showed that the majority of the items (59%) exceeded the 0.50 threshold, detecting an adequate single item reliability. Less reliable items were items 4, 5, 14, and 17 of OrNe factor, and items 1, 11 and 15 of HeOr factor. The composite reliability indicators *ρ*_c_ were 0.90 and 0.91 for HeOr and OrNe, respectively. Results showed that the *ρ*_c_ values exceeded the 0.70 threshold for both HeOr and OrNe.

Finally, with regard to test–retest reliability, the ICC was 0.84 for HeOr and 0.83 for OrNe, showing a good temporal stability of the TOS over a 2-week interval. Taken together, these results showed that individual reliability of items, test–retest reliability, and construct reliability can be considered adequate (Hp2).

### The TOS validity

**Fig. 2:**
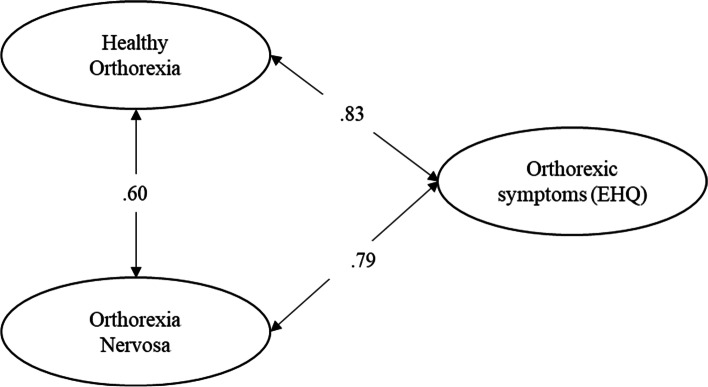
Correlations between the TOS factors and symptoms of orthorexia measured by the EHQ. Standardized solution. All parameter estimates are significant at *p* < 0.001

We hypothesized that orthorexic symptoms (EHQ) would be positively related with both HeOr and OrNe, as prior findings evidenced that EHQ items seem to tap both OrNe and HeOr dimensions [21]. Goodness-of-fit indexes indicated that the model had a good fit to the data (see Table 3). The standardized solution is shown in Fig. 2. Results showed that orthorexic symptoms (EHQ) were positively and significantly related with both HeOr (Hp3a) and OrNe (Hp3b).

**Fig. 3:**
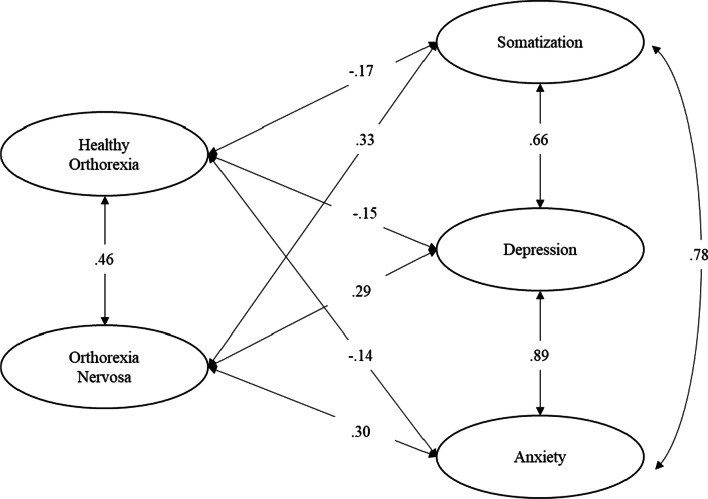
Correlations between the TOS factors and psychological distress dimensions. Standardized solution. All parameter estimates are significant at *p* < 0.001

We hypothesized that somatization, depression, and anxiety would be unrelated or negatively related with HeOr and positively related with OrNe. Goodness-of-fit indexes indicated that the model had a good fit to the data (see Table 3). The standardized solution is shown in Fig. 3. Results showed that somatization, depression and anxiety were negatively and significantly related with HeOr (Hp4a), and positively and significantly related with OrNe (Hp4b). Thus, individuals who reported higher levels of HeOr were more likely to report lower levels of somatization, depression and anxiety, while individuals who reported higher levels of OrNe were more likely to report higher levels of somatization, depression and anxiety.

**Fig. 4:**
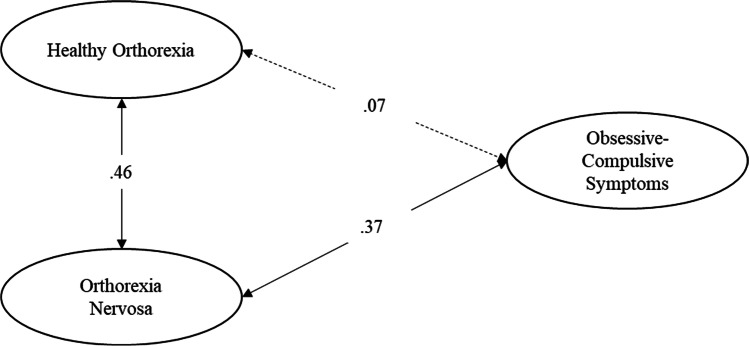
Correlations between the TOS factors and obsessive–compulsive symptoms. Standardized solution. All parameter estimates are significant at *p* < 0.001, with the exception of that represented by dashed lines

We hypothesized that obsessive–compulsive symptomatology would be unrelated or negatively related with HeOr and positively related with OrNe. Goodness-of-fit indexes indicated that the model had a good fit to the data (see Table 3). The standardized solution is shown in Fig. 4. Results showed that obsessive–compulsive symptomatology was not significantly related with HeOr (Hp5a), while it was positively and significantly related with OrNe (Hp5b). Thus, individuals who reported higher levels of OrNe were more likely to report higher levels of obsessive–compulsive symptoms.

**Fig. 5:**
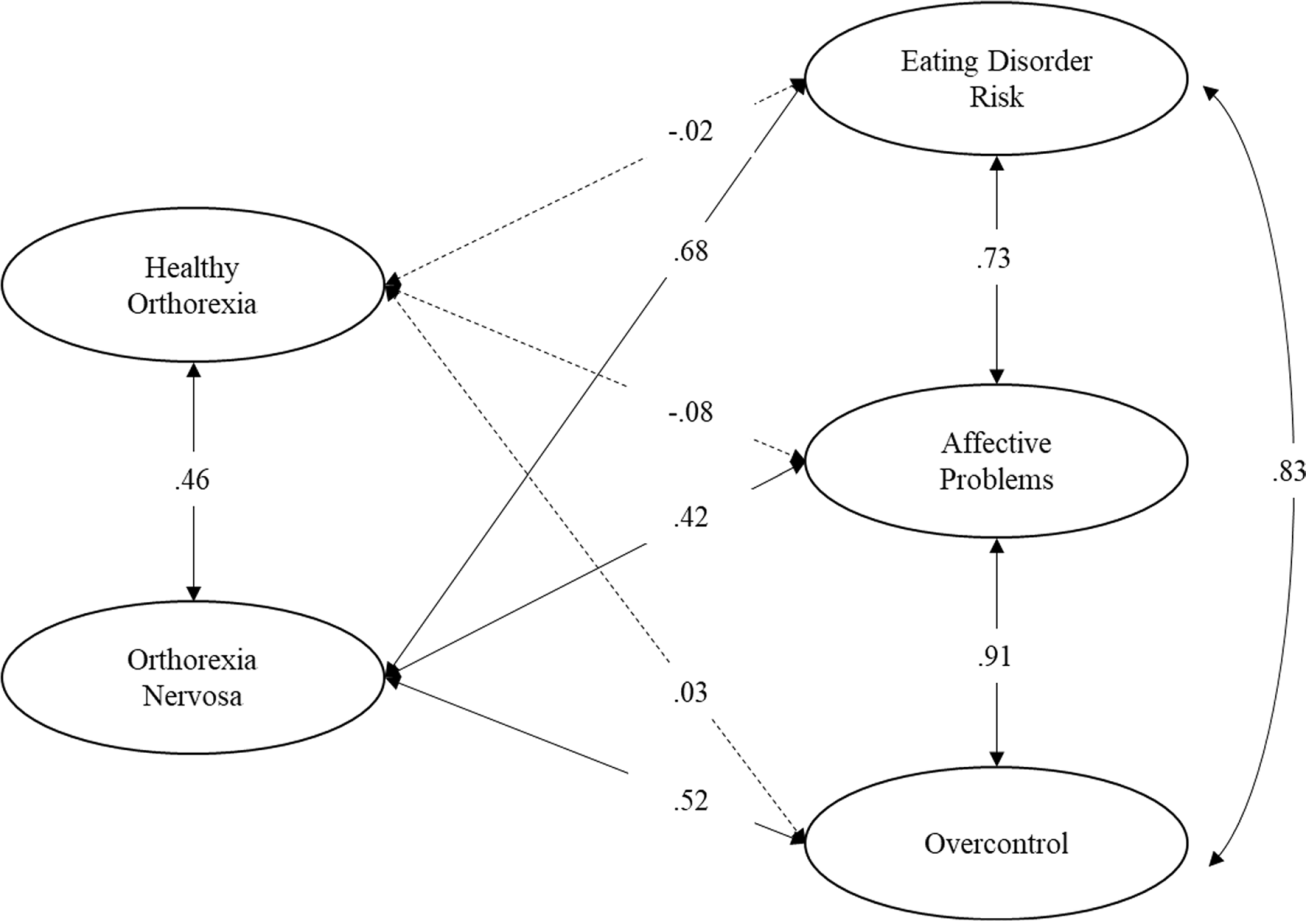
Correlations between the TOS factors and eating disorders symptoms. Standardized solution. All parameter estimates are significant at *p* < 0.001, with the exception of those represented by dashed lines

Finally, we hypothesized that Eating disorder risk, affective problems, and overcontrol would be unrelated or negatively related with HeOr and positively related with OrNe. Goodness-of-fit indexes indicated that the model had a good fit to the data (see Table 3). The standardized solution is shown in Fig. 5. Results showed that generally eating disorders symptoms were not significantly related with HeOr (Hp6a), while they were positively and significantly related with OrNe (Hp6b). Thus, individuals who reported higher levels of OrNe were more likely to report higher levels of eating disorders features.

## Discussion

The aim of this study was to examine the psychometric properties of the TOS by contributing to its Italian validation. In line with studies that have already used the TOS in other languages and countries [22, 25–29], our results indicated good psychometric properties for the Italian version of this measure, confirming the validity of the 2-correlated factors structure originally proposed by Barrada and Roncero [23]. Results provided thus evidence for the adequacy of the TOS in discriminating between a pathological preoccupation with healthy diet (OrNe), assessed by the *Orthorexia Nervosa* subscale (8 items), and a healthy interest in proper nutrition (HeOr), assessed by the *Healthy Orthorexia* subscale (9 items). With regard to the examination of standardized factor loadings, our results showed that almost every item of the TOS loaded on the supposed factor, with the exception of item 9 (“My concern with healthy eating takes up a lot of my time”) which presented a high and significant cross-loading. This is in line with the French-Canadian validation of the TOS [25], which found that item 9 presented a higher level of association with the HeOr factor rather than with the OrNe. This result may suggest that spending a lot of time on healthy eating may be seen as a non-concerning way of life, and thus should not be considered as a core feature of OrNe. Based on this evidence, it would be recommended to use the TOS by omitting item 9 in future research. With regard to the second specific aim of the study, results showed good reliability of the instrument, both in terms of internal consistency and temporal stability, confirming the study’s second hypothesis.

Regarding the associations between the TOS dimensions and the assessed variables, our results are in line with findings from previous studies [22, 23, 25–29], showing that OrNe was significantly associated with overall eating disorders risk, affective problems, overcontrol, somatization, anxiety, depression, and obsessive–compulsive symptoms, while HeOr was unrelated or inversely associated with psychopathology and emotional distress. Specifically, there was a high association between OrNe and the eating disorders risk score, which seems to support a representation of OrNe as a condition strictly related to, although differentiated from, the eating disorders spectrum [8, 51]. In this respect, also the association between OrNe and the psychological characteristics of eating disorders assessed by the EDI-3 (affective problems, overcontrol) seems to further strengthen the previously mentioned relationship. HeOr was instead unrelated to measures of eating disorders, supporting the independency of HeOr from problematic eating conditions [23]. Moreover, we found significant associations between OrNe and measures of psychopathological symptoms (somatization, anxiety, and depression), in line with previous studies that suggested that OrNe behaviors may serve as dysfunctional coping strategies to manage emotional distress [7, 52]. Conversely, the negative association found between HeOr and psychopathology may indicate that more flexibility related to healthy-eating behaviors in HeOr individuals may serve as a protective factor against emotional distress, thereby enhancing psychological well-being. It is therefore possible that the differences between OrNe and HeOr regarding relation to psychopathology are attributable to underlying differences in terms of psychological functioning, which may be characterized by a greater rigidity in OrNe individuals [7].

Finally, we assessed the correlations between both TOS subscales and orthorexic symptoms measured by another instrument, the EHQ. As expected, we found that both OrNe and HeOr were strongly and positively associated with a single dimension defined by the three factors of the EHQ. This finding strengthens what previous research has stated about the inability of the most commonly used instruments (including the EHQ) to discriminate between pathological and non-problematic forms of interest in healthy eating, suggesting the importance to refer to new and more reliable measures [4, 18].

## Strength and limits

A number of study limitations need to be addressed. First, the distribution of our participants by gender was unbalanced, with a prevalence of female participants, so it was not possible to test the TOS invariance across gender, although it has been shown in the French-Canadian and in the Portuguese–Brazilian validation studies [25, 27]. This is an issue that could bias results and limit generalizability of the findings. Future research would benefit from the replication of this study with more heterogeneous and representative groups of people. Second, we only administered self-report questionnaires, which may be sensitive to social desirability bias, possibly inflating some of the associations among variables. Future research should use a multi-method approach, including qualitative interviews. Third, it is worth noting that the study’s participants were recruited during the COVID-19 pandemic. Considering the potential effects of the pandemic on individuals’ psychological well-being, we cannot exclude that this could have interfered with our results. Finally, although the back-translation procedure is generally considered as the most used method to translate questionnaires from the original language to another, it may present some limitations due to possible translation errors and cultural biases [53].

Despite these limitations, this is the first study evaluating and supporting the psychometric properties of an Italian version of the TOS, which can be considered a useful measure for the assessment of both pathological and non-problematic forms of orthorexia in Italian samples. It may also represent a valuable instrument in clinical practice, leading to more accurate assessments and targeted interventions. Indeed, given the relevant limitations of the commonly used measures in the evaluation of orthorexic symptoms (particularly, the ORTO-15), the TOS could represent a valid and reliable choice in this area.

## What is already known on this subject?

Starting from the original Spanish version, the two-factor structure of the TOS has been tested in English-, French-. Portuguese-, Arabic-, and Turkish-speaking samples. This measure showed good psychometric properties and significant relationships with theoretically related constructs also in these contexts, highlighting the importance to distinguish between non-problematic and problematic forms of concerns with healthy eating.

## What your study adds?

The present study provided evidence for the adequacy of the TOS psychometric properties also in the Italian context, supporting the use of this measure as a valid and reliable tool to assess orthorexic behaviors in adult Italian-speaking samples.

## Data Availability

The data that support the findings of this study are available upon reasonable request from the corresponding author.
